# A Strategic Partnership to Advance AI Applications in Genomics and Bioinformatics for Health Innovation

**DOI:** 10.2196/93272

**Published:** 2026-03-27

**Authors:** Aik Choon Tan, Ece Dilber Gamsiz Uzun

**Affiliations:** 1 MidSouth Computational Biology and Bioinformatics Society (MCBIOS), Salt Lake City, UT, United States; 2 Department of Oncological Sciences and Biomedical Informatics, Huntsman Cancer Institute, University of Utah, 1950 Circle of Hope Dr, Salt Lake City, UT, 84091, United States; 3 Department of Pathology and Laboratory Medicine, Alpert Medical School, Brown University, 593 Eddy Street, Providence, RI, 02903, United States, 1 4014444000; 4 Department of Pathology and Laboratory Medicine, Brown University Health, Providence, RI, United States; 5 Center for Clinical Cancer Informatics and Data Science (CCIDS), Brown University, Providence, RI, United States; 6 Center for Computational Molecular Biology (CCMB), Brown University, Providence, RI, United States

**Keywords:** artificial intelligence, bioinformatics, genomics, AI, health innovation

## Abstract

*JMIR Bioinformatics and Biotechnology* announced a strategic partnership with the MidSouth Computational Biology and Bioinformatics Society (MCBIOS) in late 2025; this partnership establishes JMIR Bioinformatics and Biotechnology as the official journal of MCBIOS. This collaboration reflects a shared commitment to advancing computational biology, bioinformatics, and biotechnology through open science, interdisciplinary collaboration, and real-world data. By connecting MCBIOS’ community-building and professional development initiatives with JMIR Publications’ open-access publishing platform, this partnership aims to support emerging researchers, accelerate the dissemination of innovative computational methods and artificial intelligence applications, and strengthen data-driven research across medicine and biology.

In late 2025, *JMIR Bioinformatics and Biotechnology* announced a new strategic partnership with the MidSouth Computational Biology and Bioinformatics Society (MCBIOS), under which it will serve as the official journal of MCBIOS. This collaboration represents a shared commitment to advancing computational biology, bioinformatics, and biotechnology through open science, interdisciplinary collaboration, and real-world data.

MCBIOS is dedicated to advancing the fields of computational biology and bioinformatics by fostering collaboration, networking, and professional development among scientists, educators, and trainees. The society promotes interdisciplinary research, supports educational initiatives, and provides opportunities—particularly for trainees and early-career researchers to engage with peers and leaders in the field. Through annual conferences and community outreach, MCBIOS works to enhance scientific knowledge, encourage innovation, and contribute to solving complex biomedical questions [1].

The vision of *JMIR Bioinformatics and Biotechnology* is to promote high-quality, interdisciplinary research at the intersection of bioinformatics, computational biology, biotechnology and artificial intelligence (AI). The journal aims to provide an open-access platform for innovative computational methods, data-driven biological discoveries, and translational applications that bridge algorithm development with real-world biomedical questions. Emphasizing collaboration among bioinformaticians, biologists, clinicians, and data scientists, the journal seeks to foster rigorous, reproducible research that leverages emerging technologies such as AI, machine learning, and big data analytics to accelerate scientific progress and translational impact in the life sciences and medicine.

The partnership between MCBIOS and *JMIR Bioinformatics and Biotechnology* is well-timed within today’s scientific and academic landscape for several key reasons. First, computational biology and bioinformatics have entered a phase defined by large-scale data generation, AI, and translational research. Second, aligning a scientific society that fosters community, mentorship, and scholarship with a global, open-access journal amplifies both visibility and impact. Furthermore, in an era where rapid dissemination and interdisciplinary collaboration have become key components in research, this partnership creates a streamlined pathway from conference presentation and networking to peer-reviewed publication in a journal that prioritizes accessibility and methodological innovation. Together, these organizations will support emerging scholars and strengthen open, collaborative, and AI-enabled data science.

## Strengthening a Community at the Intersection of Bioinformatics, Biotechnology, and AI

MCBIOS has long served as a vibrant forum for academic researchers, industry scientists and trainees working across computational biology, bioinformatics, systems biology, machine learning, medicine and data science. This partnership offers not only a conference or a journal to showcase research, but also an integrated professional ecosystem that amplifies visibility, supports career development, and fosters innovation—making participation both professionally valuable and intellectually impactful. As part of this partnership, *JMIR Bioinformatics and Biotechnology* will work closely with MCBIOS leadership to support the society’s mission through:

- **Official society journal designation:** Being the official journal of MCBIOS reinforces *JMIR Bioinformatics and Biotechnology*’s credibility in the publishing landscape wherein researchers must identify trusted journals. This designation will provide quality, community oversight, and alignment with the interdisciplinary standards of computational biology and bioinformatics, giving authors confidence that their work will reach the right audience.
- **Annual conference-linked theme issues:** By publishing issues connected to MCBIOS annual meetings, *JMIR Bioinformatics and Biotechnology* will help capture emerging trends and high-impact research that might otherwise remain limited to conference presentations. This addresses the challenge of slow publication process and provides authors, especially early-career researchers, a pathway from presentation to peer-reviewed publication, increasing the visibility and impact of their work in a timely manner.
- **Publishing benefits for MCBIOS members:** Discounted article processing fees in reducing financial barriers to open access publishing, addressing a major concern for researchers.
- **Education and capacity building:** Through educational and career development webinars, and resources on peer review, publishing ethics, and research dissemination, *JMIR Bioinformatics and Biotechnology* and MCBIOS aim to address a gap in formal education on modern publishing practices.

These initiatives are designed to amplify the visibility and impact of work emerging from the MCBIOS community, while welcoming impactful contributions from the broader global research ecosystem to the *JMIR Bioinformatics and Biotechnology* .

## A Shared Focus: From Computational Methods to Meaningful Clinical Impact

MCBIOS and *JMIR Bioinformatics and Biotechnology* share a vision of advancing interdisciplinary data-driven research with clinical impact through collaboration, transparency, and open dissemination. Both partners are committed to fostering innovation at the intersection of bioinformatics, computational biology, biotechnology and AI while supporting emerging investigators and strengthening research communities. Together, MCBIOS and *JMIR Bioinformatics and Biotechnology* aim to provide a scholarly home for research that not only advances methods and technologies but also demonstrates meaningful applications across biomedical, clinical, population health, and AI contexts. For example, machine learning tools that improve early disease detection or risk stratification, computational approaches that predict drug response, or real-world data analyses that evaluate the viability of computational tools in clinical settings.

We invite submissions that address, but are not limited to:

- Novel computational methodologies, algorithms, and statistical approaches
- AI and machine learning applications in genomics, bioinformatics, biotechnology, and health
- Data integration and multimodal analytics, including genomics, transcriptomics, proteomics, imaging, and clinical data
- Demonstrated real-world applications in translational bioinformatics, including clinical, public health, and biotechnological use cases
- Scalable technologies and platforms enabling reproducible and open science

By emphasizing both methodological rigor and impactful applications, *JMIR Bioinformatics and Biotechnology* seeks to serve as a bridge between innovation and implementation of computational methods, supporting data-driven research that advances science while contributing to improved health outcomes.

## Looking Ahead

This partnership between *JMIR Bioinformatics and Biotechnology* and MCBIOS is not only a recognition of shared values but also an investment in the future of bioinformatics and biotechnology research. As the pace of innovation accelerates, there is a growing need for journals that can thoughtfully integrate advances in bioinformatics, data science, AI, and biotechnology within a health-focused, open access framework. We look forward to working with the MCBIOS community to shape this next chapter through collaborative theme issues, high-quality submissions, and ongoing dialogue about the evolving role of bioinformatics and biotechnology in transforming health.

We warmly invite MCBIOS members and the wider research community to submit their work to *JMIR Bioinformatics and Biotechnology* [2] and to join us in advancing research that bridges bioinformatics, data, AI, and innovation for real-world health impact.

## Abbreviations

AI: artificial intelligence
MCBIOS: MidSouth Computational Biology and Bioinformatics Society
